# Imaging features and differential diagnoses of non-neoplastic diffuse mediastinal diseases

**DOI:** 10.1186/s13244-020-00909-z

**Published:** 2020-10-15

**Authors:** Flavian Tabotta, Gilbert R. Ferretti, Helmut Prosch, Samia Boussouar, Anne-Laure Brun, Justus E. Roos, Lukas Ebner, Igor Letovanec, Michel Brauner, Catherine Beigelman-Aubry

**Affiliations:** 1 Radiodiagnostic and Interventional Radiology, CHUV-University Hospital, Rue du Bugnon 46, CH-1011 Lausanne, Switzerland; 2 Department of Diagnostic and Interventional Radiology, Grenoble Alpes University Hospital, Grenoble Alpes University, 38043 Grenoble cedex, France; 3 Department of Biomedical Imaging and Image-Guided Therapy, Medical University of Vienna, Währinger Gürtel 18-20, 1090 Vienna, Austria; 4 Radiology Department Pitié Salpetrière Hospital, 47-83 Boulevard de l’Hôpital, 75013 Paris, France; 5 Department of Radiology, Cochin Hospital, Paris Descartes University, 27 Rue du Faubourg Saint-Jacques, 75014 Paris, France; 6 Radiologie und Nuklearmedizin, Luzerner Kantonsspital, Spitalstrasse 6000, Luzern 16, Switzerland; 7 Department of Diagnostic, Interventional and Pediatric Radiology, Inselspital Bern University Hospital, University of Bern, Freiburgstrasse 18, 3010 Bern, Switzerland; 8 Institute of Pathology, CHUV-University Hospital, Rue du Bugnon 25, CH-1011 Lausanne, Switzerland; 9 Service de Radiologie, Hôpital Avicenne, 125 route de Stalingrad, 93000 Bobigny, France

**Keywords:** Diffuse mediastinal diseases, Mediastinum, Pneumomediastinum, Acute mediastinitis, Fibrosing mediastinitis and mimickers

## Abstract

Acute or chronic non-neoplastic diffuse mediastinal diseases have multiple causes, degrees of severity, and a wide range of management. Some situations require emergency care while others do not need specific treatment. Although the diagnosis may be suspected on chest X-ray, it is mainly based on CT. A delayed recognition is not uncommonly observed. Some findings may prompt the radiologist to look for specific associated injuries or lesions.

This pictorial review will successively describe the various non-neoplastic causes of diffuse mediastinal diseases with their typical findings and major differentials.

First, pneumomediastinum that can be provoked by extra- or intra-thoracic triggers requires the knowledge of patient’s history or recent occurrences. Absence of any usual etiological factor should raise suspicion of cocaine inhalation in young individuals.

Next, acute mediastinitis may be related to post-operative complications, esophageal perforation, or contiguous spread of odontogenic or retropharyngeal infections. The former diagnosis is not an easy task in the early stage, owing to the similarities of imaging findings with those of normal post-operative appearance during the first 2–3 weeks.

Finally, fibrosing mediastinitis that is linked to an excessive fibrotic reaction in the mediastinum with variable compromise of mediastinal structures, in particular vascular and airway ones. Differential diagnosis includes tumoral and inflammatory infiltrations of the mediastinum.

## Key points

- Pneumomediastinum requires exclusion of tracheobronchial injury.
- Mediastinal post-operative changes are indistinguishable from acute mediastinitis during the first weeks following surgery.
- CT, 18F-FDG PET/CT, and diffusion-weighted or dynamic contrast-enhanced MRI may help to narrow the differential diagnosis and may guide tissue sampling.

## Background and technical approach

Diffuse mediastinal diseases comprise a heterogeneous group of entities characterized by various degrees of severity [1]. Although these diseases are well-known disorders, diagnosis is not uncommonly delayed due to nonspecific and sometimes faint clinical presentations and symptoms, even in the potentially more serious conditions. Pneumomediastinum should be suspected in case of chest pain, dyspnea, and subcutaneous emphysema. However, acute mediastinitis commonly presents with fever, pleuritic or retrosternal chest pain, and inflammatory syndrome. In fibrosing mediastinitis, the main clinical symptoms include dyspnea and superior vena cava syndrome due to external vascular stricture and airway stenosis. Careful clinical evaluation and radiological correlation is of the utmost importance when approaching diffuse mediastinal pathologies in order to differentiate neoplastic mediastinal infiltration.

Although mediastinal involvement may be suspected on chest radiographs, the signs may be easily missed; hence CT, and, in selected patients, MRI, should be conducted to evaluate the mediastinal compartments. High-resolution imaging is required to provide a detailed assessment of the mediastinal compartment and its complex anatomy, as well as associated anomalies. CT, with a water-soluble contrast agent following a non-contrast acquisition, may demonstrate the esophageal rupture in case of pneumomediastinum, or stenosis in case of chronic mediastinitis. Next, an acquisition with IV contrast may be beneficial in acute and chronic mediastinitis—simultaneous injection of contrast material via brachial veins on both sides may be considered—because this can also depict the regional environment, as well as any additional findings. 18F-FDG PET/CT and diffusion/dynamic contrast-enhanced MRI [2] may be helpful in assessing disease extent and guiding tissue sampling. Fluoroscopy may be part of the diagnosis if an esophageal rupture is suspected and/or in case of vascular compromise as part of the therapeutic procedure.

Specific associated injuries or lesions need to be systematically determined, based on the imaging findings. Various non-neoplastic conditions will be discussed in the following sections.

## Clinical scenarios

### Pneumomediastinum

#### Pathophysiology

**Fig. 1:**
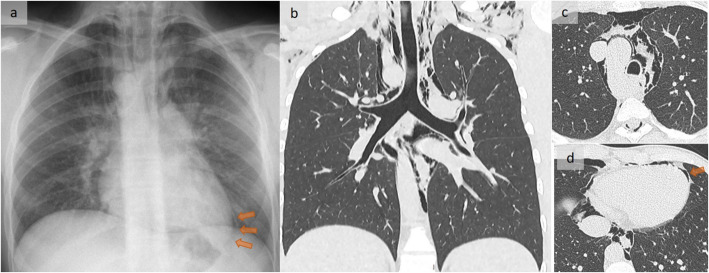
Atraumatic pneumomediastinum. A patient with sudden spontaneous retrosternal and interscapular pain. Frontal chest X-ray (**a**), coronal oblique reformation in lung windowing (**b**), and axial slices (**c**, **d**) show gas outlining the mediastinal structures, with pneumopericardium (orange arrows in **a** and **d**) appearing as a left paracardiac subtle line in **a** and subcutaneous emphysema. Note the right aortic arch. Conservative management led to a favorable outcome

**Fig. 2:**
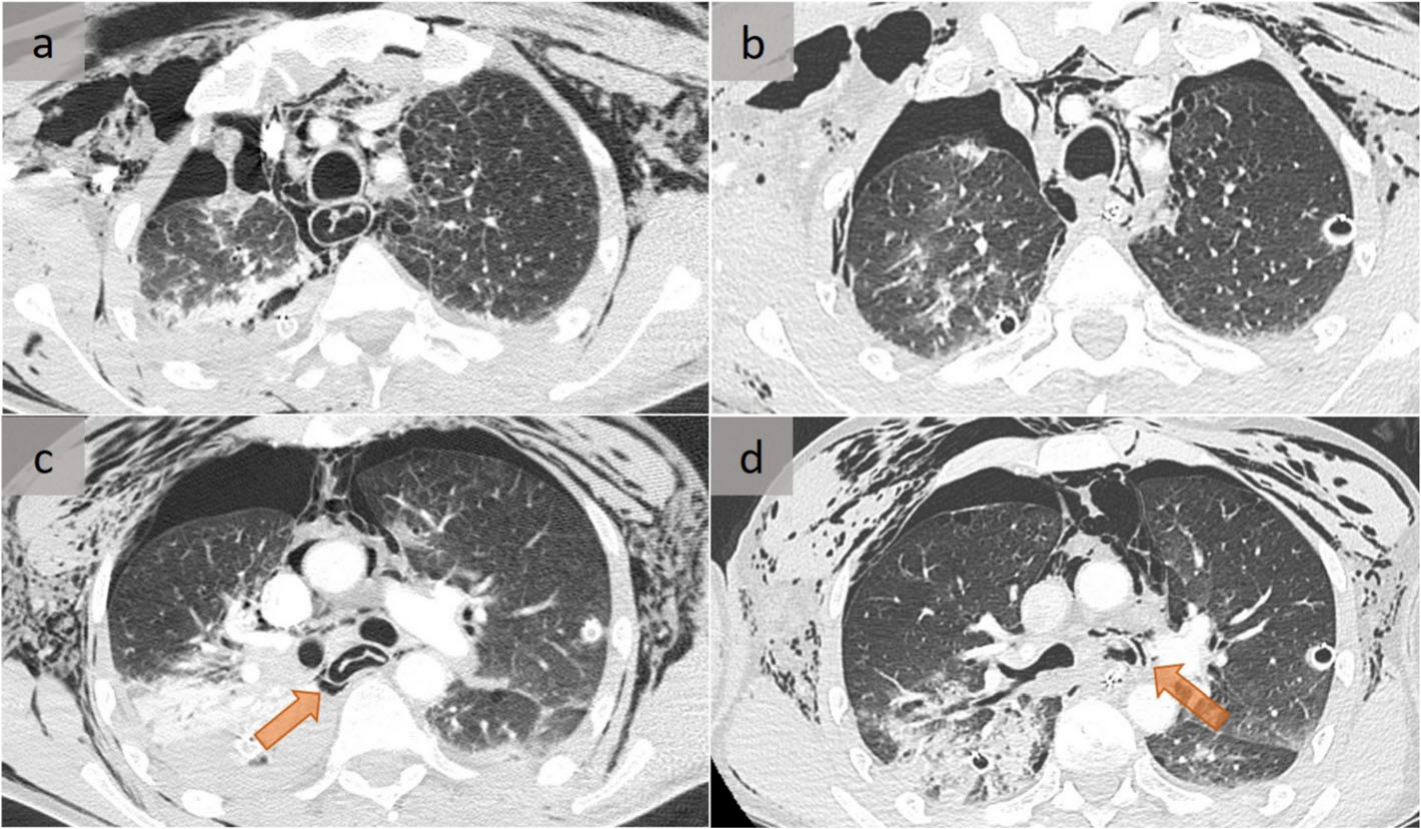
Traumatic pneumomediastinum. A patient who experienced high-velocity trauma while skiing with subcutaneous emphysema, bilateral pneumothorax, and pneumomediastinum. Note the air within the esophageal wall that subsequently resolved 1 day later, at the level of the supra-aortic vessels (**a**, **b**) and below the carina (**c**, **d**) (orange arrows). This appearance mimics the presence of an intramural esophageal dissection that mandates the need to exclude an esophageal injury, which was the case here. Repeated bronchoscopy performed due to persistent air leak through the right drainage tube revealed multi-segmental right bronchial fistulae

Pneumomediastinum, defined as free gas located within the mediastinum, may dissect along fascial sheaths (Fig. 1) to create a cervical and thoracic subcutaneous emphysema [3]. Pneumorrachis, pneumoperitoneum, or rarely, retropneumoperitoneum, and even air within the esophageal wall that mimics an esophageal dissection (Fig. 2), may be observed [4].

**Fig. 3:**
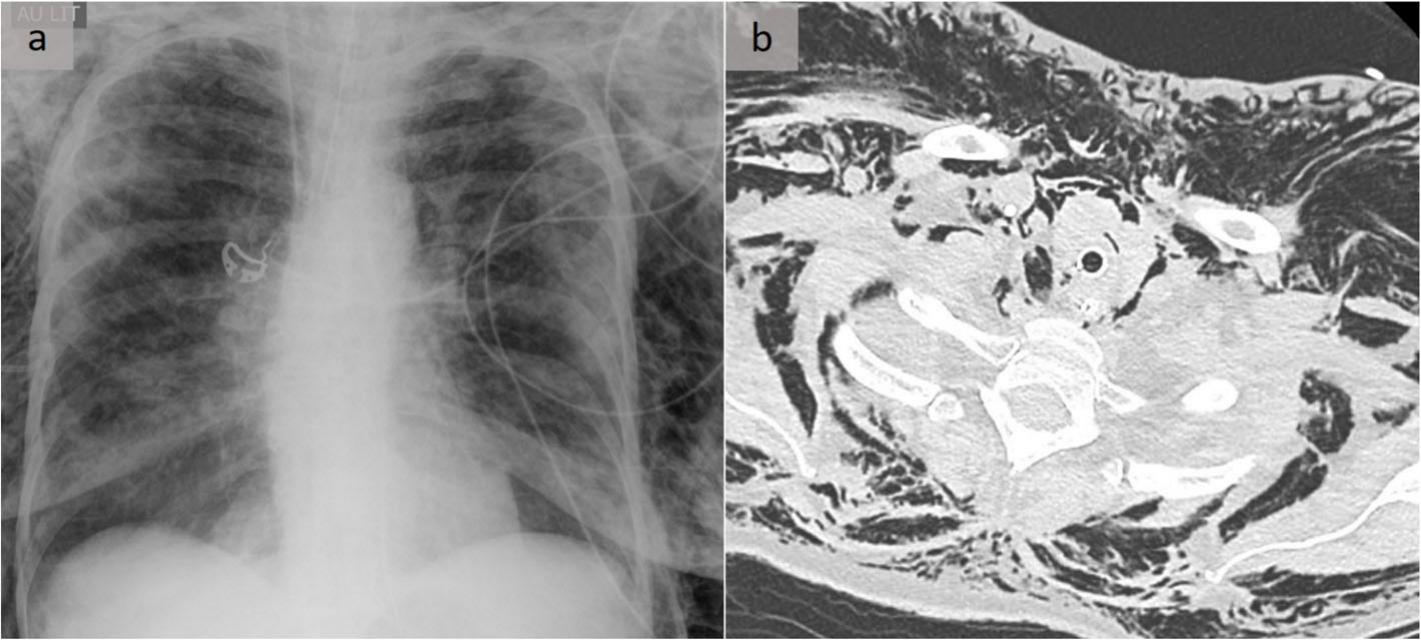
Pneumomediastinum related to tracheo-esophageal injuries. A patient resuscitated after cardiac arrest with pneumomediastinum related to both tracheal and esophageal injuries. The major finding on the chest X-ray (**a**) was the subcutaneous emphysema. Conversely, the pneumomediastinum is perfectly demonstrated on the axial CT scan (**b**)

The Macklin effect, or pulmonary interstitial emphysema, which is responsible for more than 95% of cases, corresponds to an alveolar rupture with air dissection along the interstitial sheaths toward the mediastinum (Fig. 1), eventually leading to a pneumomediastinum [5]. The Macklin effect, more frequent than esophageal or tracheobronchial disruptions, has been reported in 39% of cases of severe, blunt trauma pneumomediastinum. It has been associated with a significantly prolonged intensive care stay, reflecting severe trauma [3, 5]. Importantly, its identification does not rule out a tracheobronchial injury (Fig. 2), which must be excluded in cases of traumatic as well as post-procedure pneumomediastinum [4] (Fig. 3).

#### Iatrogenic causes

At least half of all esophageal perforations are estimated to be iatrogenic in nature. This can result from endoscopic or surgical procedures, as well as thermal injury during left atrial ablation. Typical imaging findings include pneumomediastinum, pleural effusion on the left side more commonly than on the right side, and mediastinal hematoma [6] with extravasation of swallowed water-soluble contrast.

#### Boerhaave’s syndrome

**Fig. 4:**
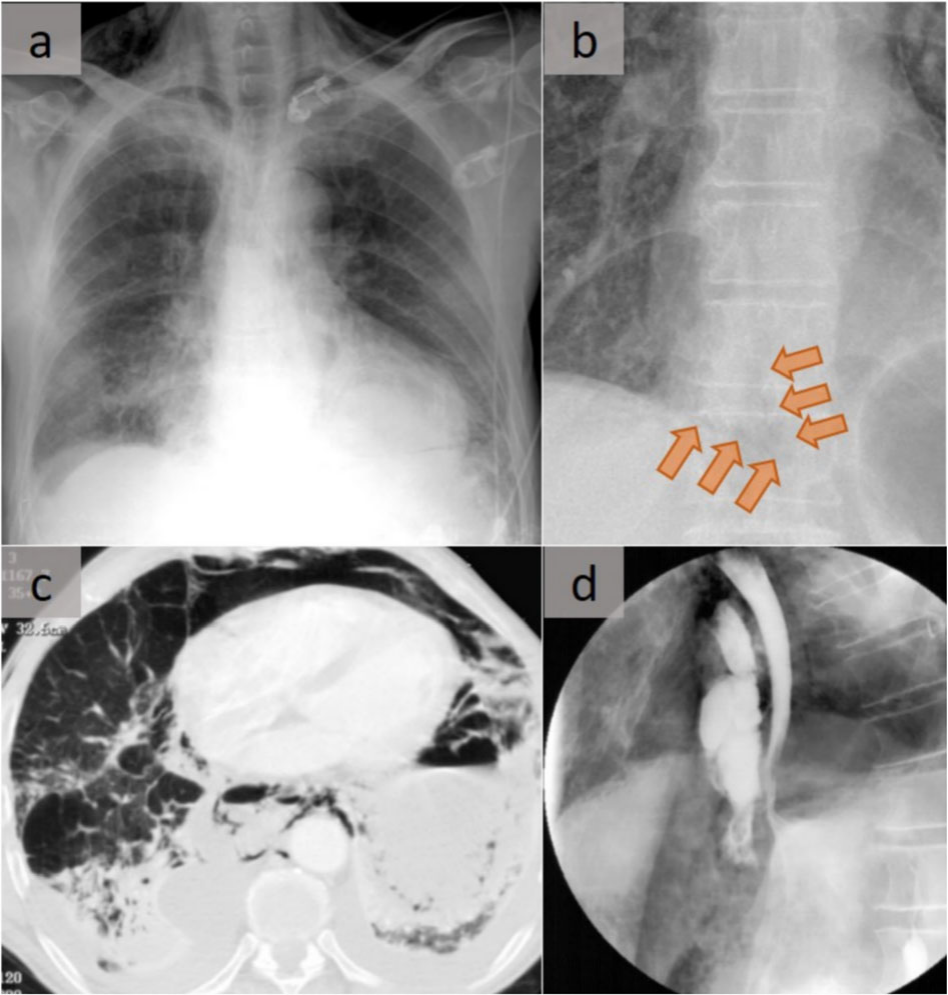
Boerhaave’s syndrome related to esophageal rupture. **a** Chest X-ray shows pneumomediastinum with subcutaneous emphysema. **b** Naclerio V sign in another patient. In this case, the Naclerio V sign was the only finding that raised the suspicion of an esophageal rupture (not shown) with a focal and limited pneumomediastinum. **c** Axial CT scan in lung windowing that shows the pneumomediastinum with bilateral pleural effusion and alveolar consolidation (**d**). Iodine hydro-soluble contrast on a lateral view shows extravasation above the diaphragm in front of the esophagus (Patient (a))

Spontaneous rupture, known as Boerhaave’s syndrome, occurs when incomplete crico-pharyngeal relaxation during vomiting results in a sudden increase in intraluminal esophageal pressure (Fig. 4). Rupture is most common in the distal left posterior wall immediately above the diaphragm. It may be suspected on a chest X-ray in patients with esophageal rupture but is not specific to that condition. Esophageal rupture is characterized by a V-shaped air collection, with one limb of the V produced by mediastinal gas that outlines the left lower lateral mediastinal border, and the other limb produced by gas between the parietal pleura and medial left hemidiaphragm. Mortality is high without prompt thoracotomy [6].

#### Tracheobronchial lesions

Tracheobronchial lesions are rare, occurring in 1 to 3% of patients with blunt trauma and 2 to 9% of those who suffer penetrating cervical and/or thoracic injuries. The former preferentially causes lesions in the intra-thoracic trachea and main-stem bronchus, 80% of which are up to 2.5 cm from the carina, most commonly on the right side, and are considered less protected by the mediastinal structures. Uncommon causes include iatrogenic injuries, such as intubation-related trauma [7] (Fig. 3).

#### Other conditions

Other causes of pneumomediastinum include acute exacerbation of severe asthma, excessive coughing, diabetic ketoacidosis, physical exertion, the Valsalva maneuver, or inhalational drug abuse [5], as well as extra-thoracic causes, such as facial fracture. In young individuals, the absence of other etiological factors should raise the suspicion of free-base cocaine use [8]. Rare circumstances, such as a lightning strike or insufflation of the content of a dry chemical fire extinguisher, have also been reported.

### Acute mediastinitis

#### Pathophysiology

Most cases of acute mediastinitis are encountered following cardiovascular or other thoracic surgical procedures. Risk factors for post-sternotomy mediastinitis may be categorized into patient-related, including age as well as co-morbidities, such as obesity, diabetes, history of smoking, COPD, and renal failure, and operative factors or environmental elements (operating room and medical devices) [9].

**Fig. 5:**
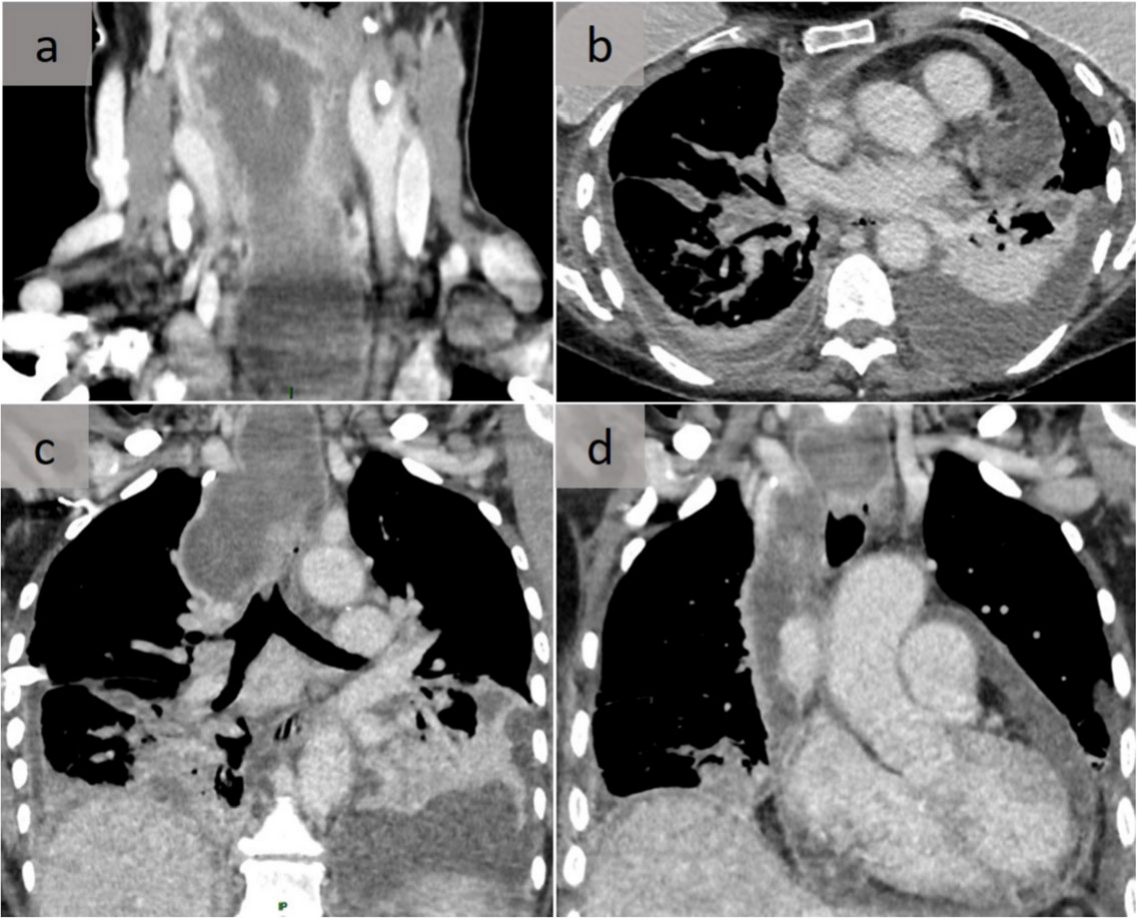
Infectious mediastinitis. Acute, descending, necrotizing mediastinitis related to an oropharyngeal infection treated with non-steroidal anti-inflammatory drugs and antibiotics. Latero-pharyngeal (**a**) and median and right-sided mediastinum are (**c**) hypodense, with peripheral enhancement collections in coronal reformatting and bilateral pleural effusion and passive atelectasis (**c**); pleural effusion with parietal pleural enhancement related to bilateral empyema and pericardial effusion, and parietal pericardial enhancement related to purulent pericardial effusion on axial (**b**) and coronal views (**d**)

Other cau ses typically comprise either esophageal perforation or the contiguous spread of odontogenic or retropharyngeal infections (Fig. 5). Rarely, mediastinitis may result from a penetrating trauma or hematogenous spread of infection.

#### Imaging findings

**Fig. 6:**
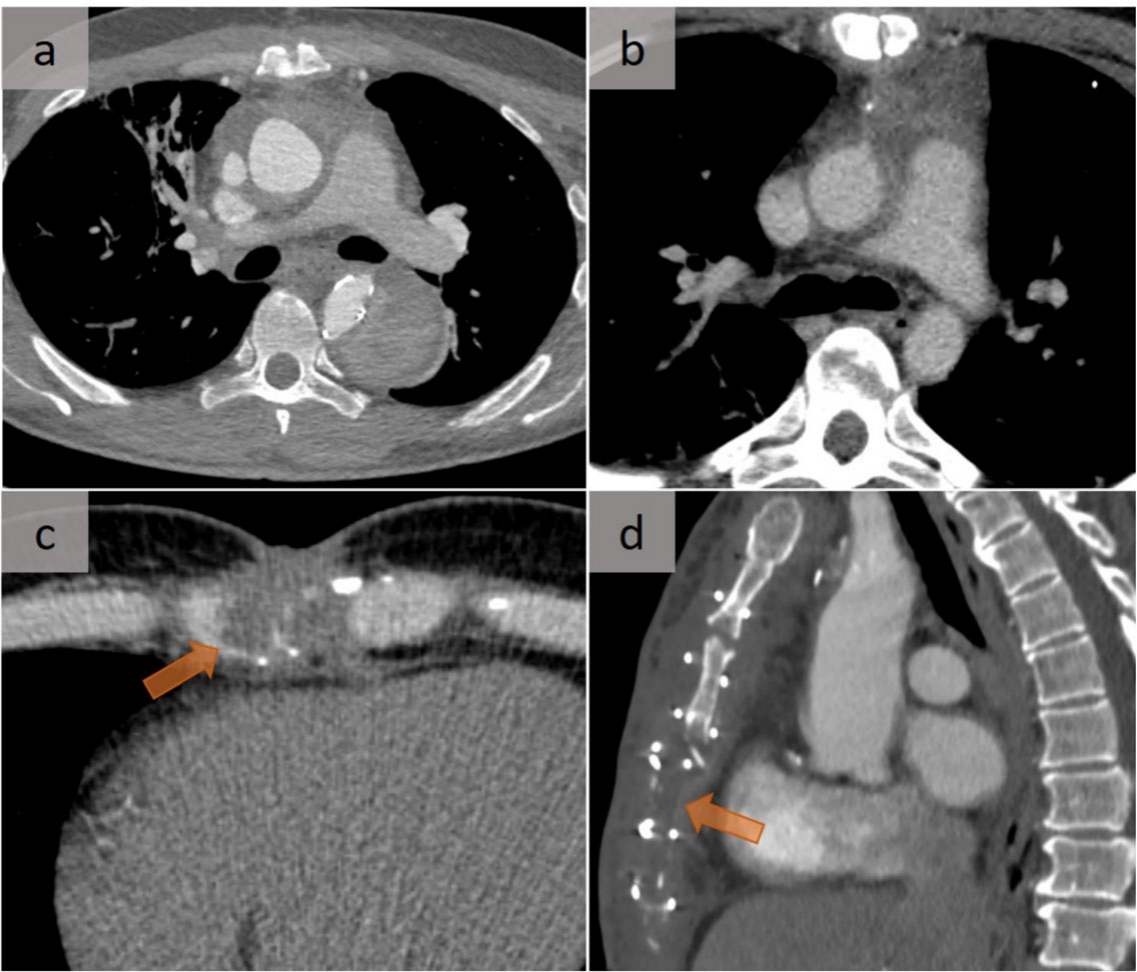
Post-operative changes versus mediastinitis. Similarities of CT aspects in two different conditions: normal post-operative appearance of the mediastinum during the first 2–3 weeks after Bentall surgery (**a**) and mediastinitis (**b**), both presenting as an increased attenuation of fat. In addition, the careful analysis of bone and cartilage, which is required in all cases of acute mediastinitis using bone-windowing, is well demonstrated in these two different cases with cartilage abscess (**c**) and sternal osteitis (**d**), respectively

In a retrospective study that included 40 various causes of acute mediastinitis, the most common abnormal findings were represented by increased attenuation of fat and pleural effusion, followed by free gas bubbles in the mediastinum, localized mediastinal fluid collections, sternal dehiscence, mediastinal lymph nodes, pericardial effusion, lung infiltrates, and pleuro-mediastinal fistula [10]. There was a high sensitivity and specificity for CT after day 17 in post-operative patients, with a low diagnostic yield in the early post-operative period. The normal post-operative appearance of the mediastinum during the first 2–3 weeks after surgery may mimic mediastinitis (Fig. 6) and may not return to normal for as long as 2 months after surgery. This lack of specificity of CT during the first 2 weeks requires a careful correlation between clinical and radiological findings that require contrast-enhanced CT.

In any case, careful analysis of cartilage and bone is necessary to detect loco-regional involvement, such as a cartilaginous abscess or focal osteitis (Fig. 6).

#### Complications

**Fig. 7:**
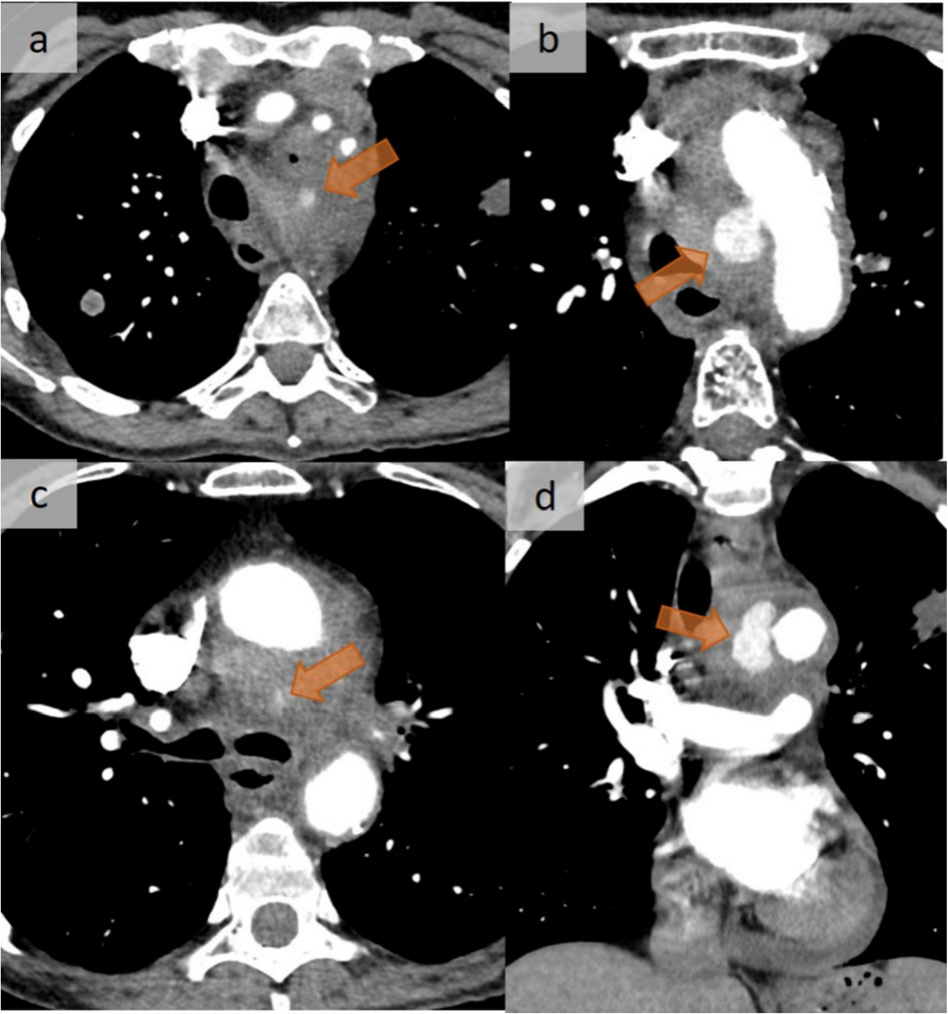
Post-mediastinitis aneurysm. Fatal, false aneurysm complicating a mediastinitis following an EBUS procedure. EBUS had been performed in the context of a suspicion of stage IV lung cancer. Contrast-enhanced CT was performed 35 days after EBUS because the patient complained of chest pain. Successive axial CT images (**a**–**c**) show a diffuse infiltration of the mediastinal fat with a false aneurysm (arrow) that developed on the right side of the aortic arch. Coronal reformation shows the cranio-caudal extent of the false aneurysm (arrow) (**d**). A pericardial effusion is present

Fatal aortic arch ruptures (Fig. 7) have been reported in the context of mediastinitis that originated from several conditions, including odontogenic infection, foreign body ingestion, endoscopic botulinum toxin injection, or after open heart surgery [11].

### Fibrosing mediastinitis and mimickers (Table 1)

#### Pathophysiology

Fibrosing mediastinitis (FM), also known as sclerosing mediastinitis, is a condition related to an abnormal immunologic reaction that results in the proliferation of fibro-inflammatory tissue in the mediastinum and/or hila [12]. It leads to a variable constriction of systemic veins, pulmonary arteries and veins, the airways and/or the esophagus, with a morbidity related to location and extent of fibrosis. The pleura, pericardium, and coronary arteries may also be involved [12].

**Table 1:** Fibrosing mediastinitis and differential diagnoses

Clinical scenarios		DIAGNOSIS				DIFFERENTIAL DIAGNOSIS		
		Fibrosing mediastinitis				Lymphoma	Castleman disease	Sarcoidosis
		Granulomatous subtype	Nongranulomatous subtype	Erdheim-Chester	IgG-4			
**IMAGING FEATURES OF FIBROSING MEDIASTINITIS AND DIFFERENTIAL DIAGNOSIS**	**X-ray Features**	Focal calcified paratracheal, subcarinal or hilar mass ++ Widening of the mediastinum Distortion and obliteration of lines				Mediastinal widening Retrosternal filling Hilar enlargement	Mediastinal masses Displacement of adjacent structures Ipsilateral pleural effusion Periostal reaction	Mediastinal widening Mass effect Hilar lymphadenopathies Nodal and lung calcifications Reticulonodular opacities, lung fibrosis, traction bronchiectasis
	**CT Features**	Focal calcified paratracheal, subcarinal or hilar mass ++ Superior vena cava syndrome	Infiltrating soft tissue, rarely calcified Variable heterogeneous enhancement Pulmonary arterial hypertension and bronchial arteries hypertrophy Unilateral pulmonary oedema, lung volume loss, chronic post-obstructive pneumonitis, bronchiectasis	Periaortic infiltration extending to the pericardium, right coronary sulci and/or myocardium of the right atrium with pleural involvement Thickening of the peribronchovascular bundles and interlobular septae	Diffuse mass in the posterior mediastinum	Superior vena cava syndrome Lymphadenopathies, including internal mammary, axillary Pericardial, pleural (unilateral) effusion Calcification (post-treatment) Pulmonary nodule, mass-like consolidation, infiltrates	Solitary or multicentric infiltrative mediastinal mass, arborising calcifications Intense homogeneous enhancement and washout Lymph nodes Centrilobular nodules	Lymphadenopathies Perilymphatic nodules, micronodules of upper/mid lung distribution, lung fibrosis
	**MR Features**	Heterogeneous T1 signal isointense to muscle Variable T2 signal Heterogeneous enhancement post-Gadolinium injection					Iso to hyperintense T1 relative to skeletal muscle Arborising calcifications as low T2 Enhancement post-Gadolinium injection Low ADC values	
**CLINICAL AND IMAGING FEATURES OF EXTRA-THORACIC MANIFESTATIONS**	**Clinical Features**	Superior vena cava syndrome	Non specific	Non specific	Riedel’s thyroiditis Retroperitoneal fibrosis Sclerosing cholangitis Autoimmune pancreatitis	+/- Palpable lymphadenopathies +/- Hepatosplenomegaly	Fluid retention	50% asymptomatic Dry eyes Erythema nodosum Parotid enlargement
	**Imaging Features**		+/- retroperitoneal fibrosis	Bone pain Focal neurological involvement Exophthalmos Retroperitoneal fibrosis Hypophysal changes related to diabetes insipidus	CNS involvement Orbital pseudotumor Riedel thyroiditis Retroperitoneal fibrosis Autoimmune pancreatitis Sclerosing cholangitis			

#### Granulomatous subtype

The most common form is the focal or granulomatous subtype of fibrosing mediastinitis, mainly due to histoplasmosis (less frequently tuberculosis), and other fungal or inflammatory conditions, such as sarcoidosis. This form usually manifests as a localized, calcified mass in the paratracheal or subcarinal regions of the mediastinum or in the pulmonary hila, commonly responsible for a superior vena cava syndrome. Diagnosis can be made based on imaging findings, preventing the need for tissue sampling.

#### Non-granulomatous subtype

**Fig. 8:**
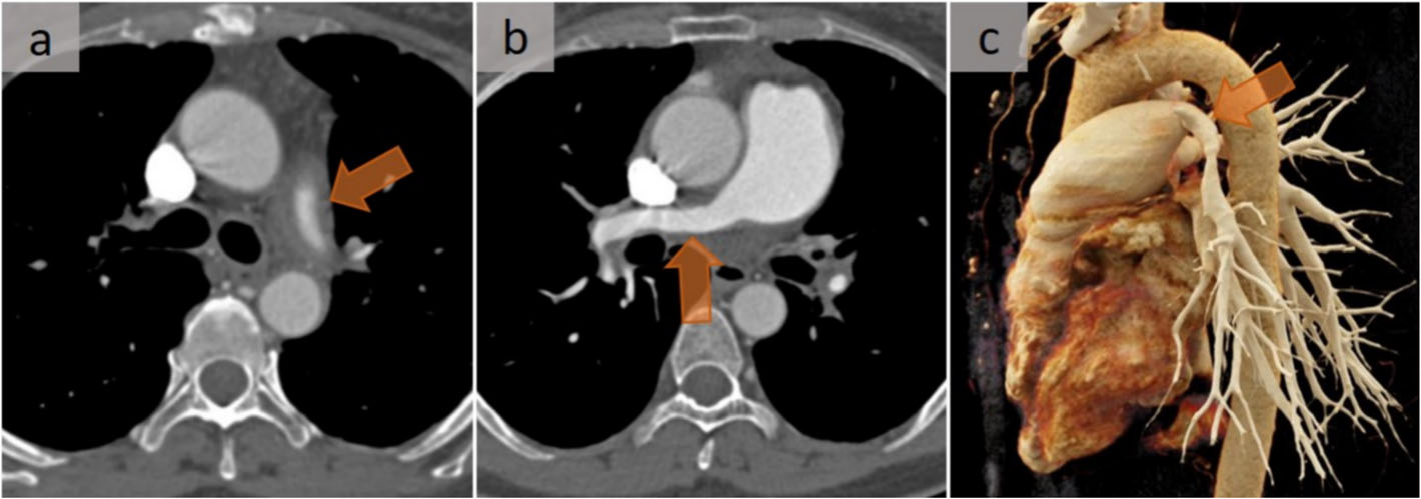
Post-mediastinitis pulmonary hypertension. Pulmonary artery stenosis in a case of fibrosing mediastinitis with pulmonary hypertension. Contrast-enhanced CT scan demonstrated at two successive levels (**a**, **b**), with a soft tissue attenuation mass diffusely infiltrating the mediastinum with encasement and narrowing of the pulmonary arteries (arrows). The stenosis of the right pulmonary artery is perfectly observed on the oblique 3D rendering view (**c**) (arrow). This mass had no significant metabolic uptake on 18F-FDG PET/CT (not shown). Note also the stenosed upper pulmonary veins

**Fig. 9:**
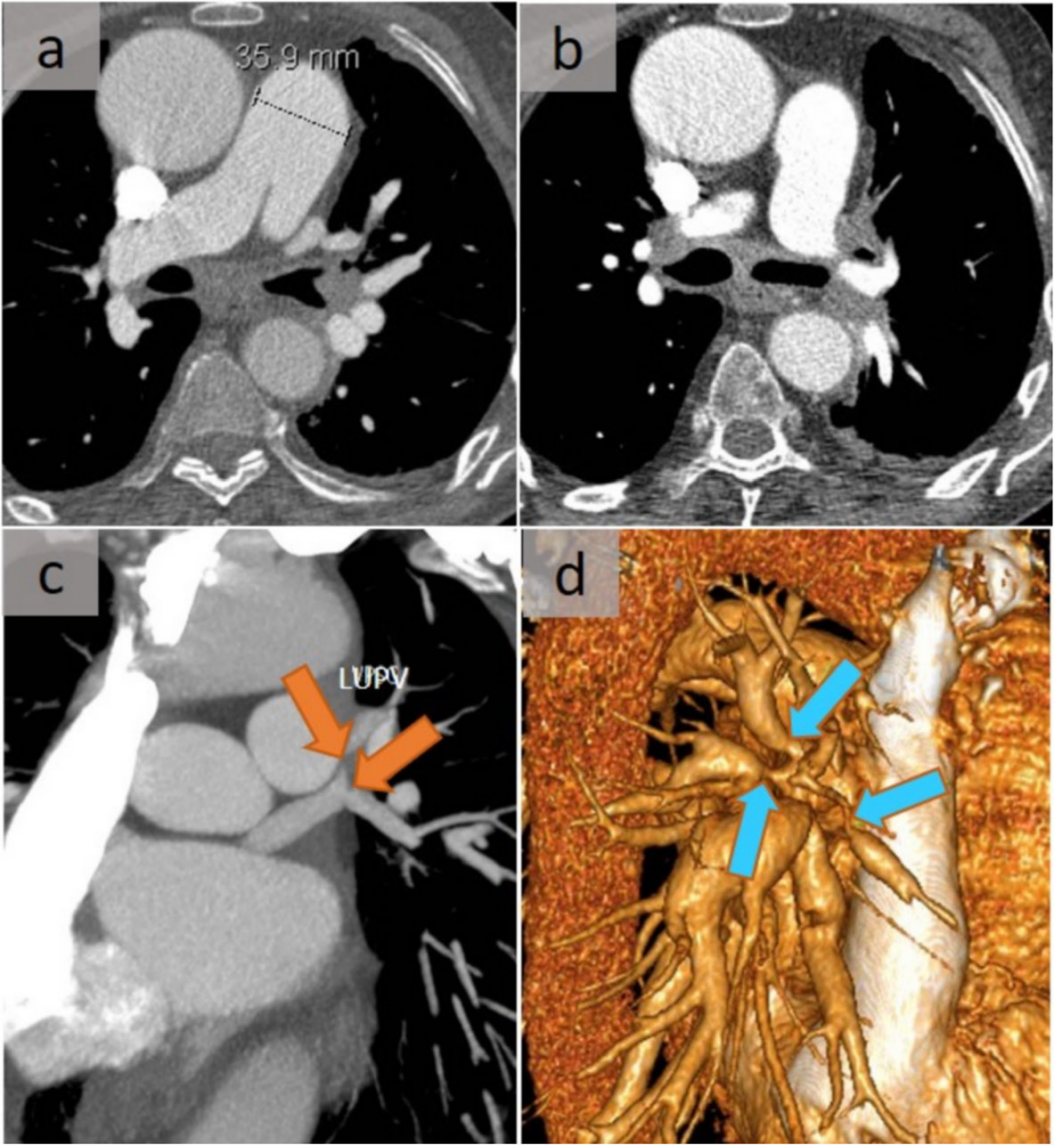
Post-mediastinitis venous complication. A patient with sclerosing mediastinitis. In addition to the encasement of the pulmonary artery, there was a narrowing of the pulmonary veins, well assessed on the successive CT slices (**a**, **b**), as well as on the oblique reformation (orange arrows) (**c**) and 3D view (blue arrows) (**d**). Diagnosis was delayed due to confounding factors related to a history of pulmonary embolism with pulmonary hypertension and tuberculosis

**Fig. 10:**
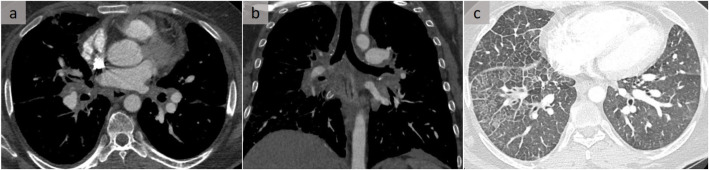
Post-tuberculous mediastinitis. A patient with chronic mediastinitis related to sequelae after tuberculosis. Axial (**a**) and coronal (**b**) CT in mediastinal windows show infiltration of the mediastinum and hila, which led to narrowing of the pulmonary veins on the right side. CT scan at the level of the heart in pulmonary windows shows right-sided septal-line thickening linked to impaired venous return (thanks to Prof. Halimi, Georges Pompidou Hospital, France)

The diffuse or non-granulomatous subtype accounts for about 10 to 20% of cases of fibrosing mediastinitis. It may be idiopathic or can originate in association with autoimmune disorders, methysergide exposure, or prior radiation exposure [12]. Fibrosing processes in other locations, such as retroperitoneal fibrosis, may also be encountered [12]. This subtype, which affects multiple mediastinal compartments and which is sometimes diagnosed after considerable delay, manifests as diffusely infiltrated soft tissue, rarely calcified, with heterogeneous enhancement after intravenous administration of contrast material. Each structure can be surrounded, encased, and/or occluded. It is noteworthy that a sequential occurrence is observed in this setting, with a primary involvement of the pulmonary veins, then the pulmonary arteries, and finally the airways. While extrinsic compression of the pulmonary arteries and/or veins is a major cause of pulmonary hypertension (Figs. 8 and 9), longstanding pulmonary venous occlusion may also lead to secondary pulmonary arterial hypertension and cor pulmonale. In addition, arterial and venous compression may lead to an imaging appearance of unilateral pulmonary edema (Fig. 10) [13], and hemoptysis may occur due to hypertrophy of the systemic arteries. With regard to airway involvement, the trachea and the main, lobar, and/or segmental bronchi may be involved and are characterized by volume loss, chronic post-obstructive pneumonitis, endoluminal mucus plugs, or bronchiectasis [12]. Although surgical biopsies are more appropriate, the role of percutaneous CT-guided core tissue biopsies should be emphasized.

#### IgG-4 related disease

**Fig. 11:**
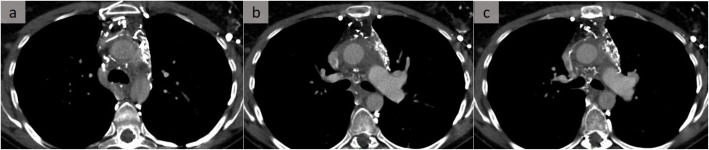
IgG4-related mediastinitis. A patient with fibrosing mediastinitis as an unusual mediastinal manifestation of IgG4-related disease. Three successive axial slices (**a**–**c**) show diffuse infiltration of the mediastinum with collaterals due to occlusion of the systemic veins

Fibrosing mediastinitis has been reported as an unusual mediastinal manifestation of IgG4-related disease (Fig. 11) [14]. The diagnosis requires a comprehensive evaluation for other disease manifestations, including Riedel’s thyroiditis, retroperitoneal fibrosis, sclerosing cholangitis, or autoimmune pancreatitis [12], which may precede and/or suggest the diagnosis. Improvement with corticosteroid therapy has been described in this setting in contrast to numerous other forms of fibrosing mediastinitis.

#### Erdheim-Chester disease

**Fig. 12:**
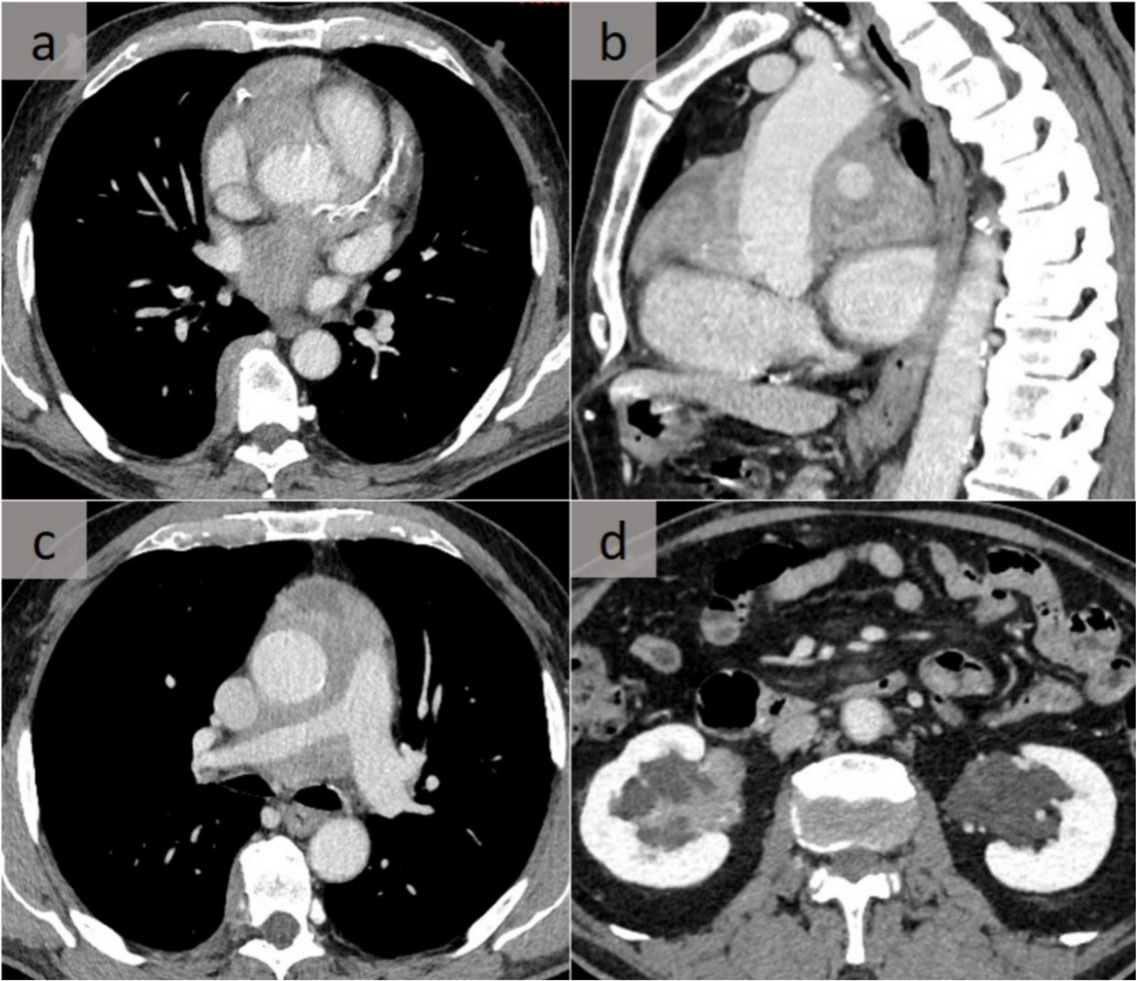
Erdheim-Chester disease. Typical pattern of Erdheim-Chester disease with infiltration of the right coronary artery sulcus (**a**) and the pericardium, well assessed on a sagittal reformation (**b**). In addition to the mediastinal/pericardial involvement encasing the right pulmonary artery, note the right paravertebral thickening on axial slices (**a**, **c**). Renal involvement with infiltration of the sinuses was also observed (**d**)

In addition to IgG4 disease, some disorders, such as Erdheim-Chester disease, must be suspected in case of multisystemic involvement. This rare, non-inherited, non-Langerhans form of histiocytosis, commonly with a BRAF mutation, is characterized by xanthomatous infiltration of the involved tissues with foamy histiocytes surrounded by fibrosis, and appears with heterogeneous systemic manifestations. A common peri-aortic infiltration that extends to the pericardium, the right coronary sulci, and/or the myocardium of the right atrium with pleural involvement may point toward this rare entity. Furthermore, bone, renal, and retroperitoneal involvement (Fig. 12) [15] have been observed. Cardiac-gated CT is required to detect coronary involvement.

#### Differential diagnosis

**Fig. 13:**
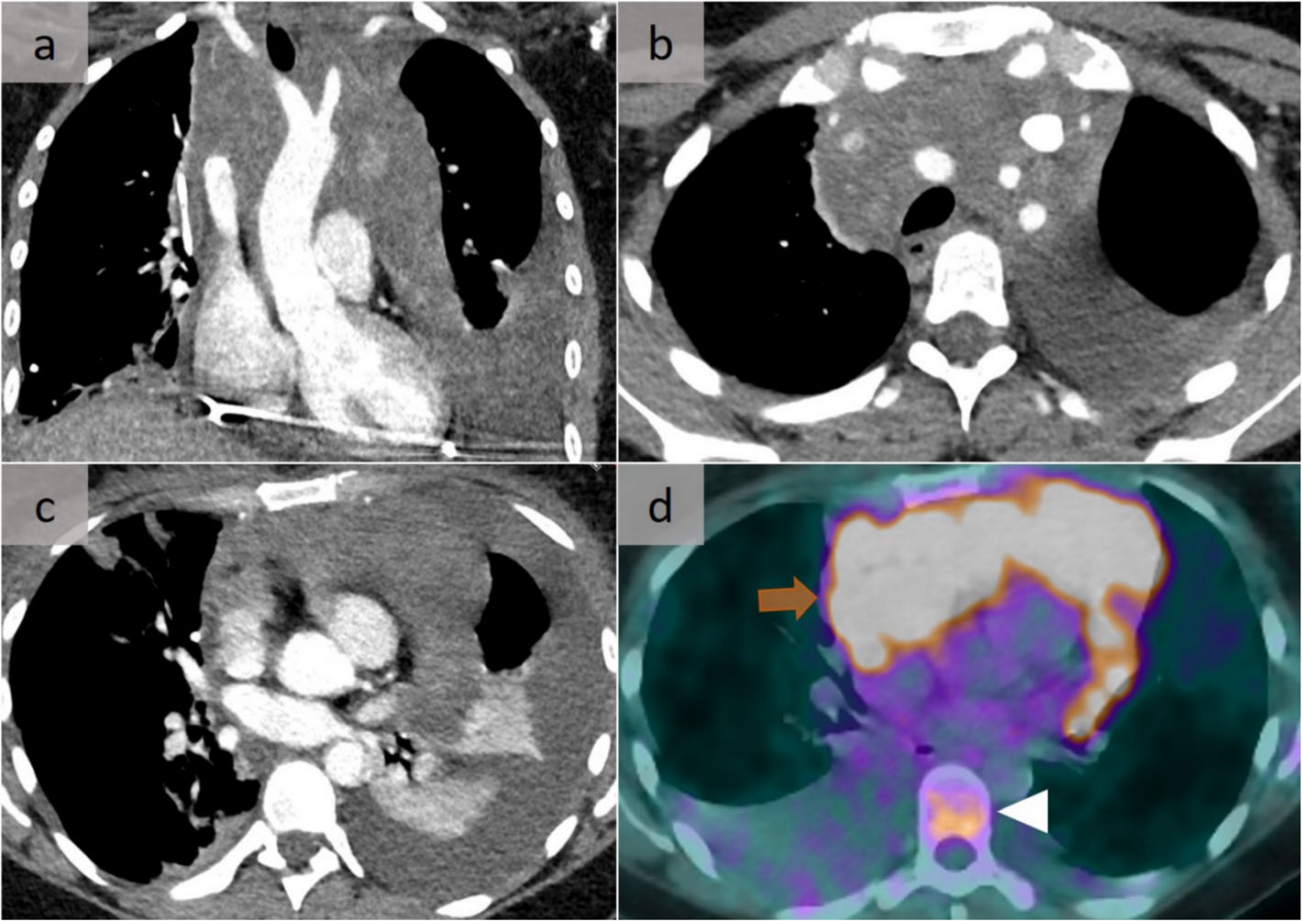
Hodgkin’s lymphoma. Diffuse mediastinal involvement well demonstrated on a coronal (**a**) and two successive axial slices (**a**, **b**). There is a contiguous pericardial involvement (**c**), with increased metabolic activity on the 18F-FDG PET-CT (**d**) (arrow). Note also the increased metabolic activity of the D11 vertebrae, suspicious for bone involvement (arrowhead), the partial resolution of the left pleural effusion following drainage, as well as a new right pleural effusion

**Fig. 14:**
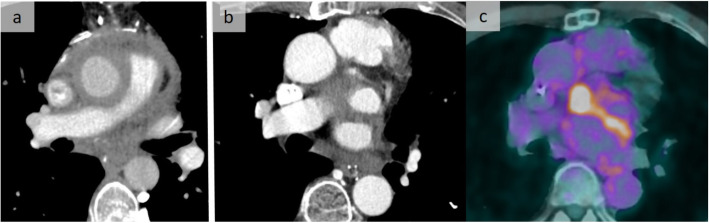
Thymoma. Two cases of diffuse infiltration of the mediastinum from different causes. The first is related to a fibrosing mediastinitis (**a**), while the other corresponds to a recurrence of a histologically proven thymoma stage IVA (**b**), highly metabolic on PET/CT (**c**). Neither disorder can be differentiated morphologically

**Fig. 15:**
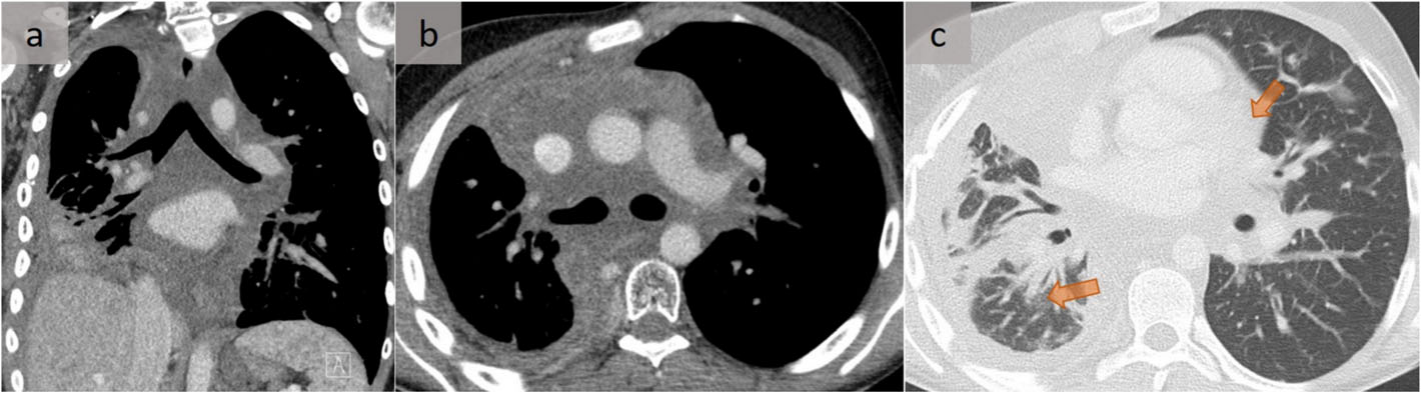
Pulmonary lymphangiomatosis. A patient with diffuse pulmonary lymphangiomatosis. Such an infiltration that may not be differentiated from another diffuse, soft tissue mediastinal disease is well assessed on a coronal (**a**) and an axial (**b**) slice. Note the associated right pleural involvement (**a**–**c**), with thickening of the pulmonary peribronchovascular bundles and interlobular septae (arrows in **c**) that reflect lymphatic distribution

The radiological differential diagnosis of diffuse fibrosing mediastinitis is extensive (Fig. 14) [16] and includes lymphoma (Fig. 13) [11], primary lung cancer, metastases, Castleman disease, and uncommonly, atypical sarcoidosis or granulomatosis with polyangiitis. Benign disorders, such as amyloidosis or lymphangiomatosis, may also diffusely infiltrate into the mediastinum (Fig. 15) [17]. The latter affects the lymphatic channels from the mediastinum to the pleura, with an associated thickening of the pulmonary peribronchovascular bundles and interlobular septae, reflecting the lymphatic distribution. Infectious disorders, such as actinomycosis, aspergillosis, zygomycosis, coccidioidomycosis, nocardiosis, mycobacterial infection, or syphilis, should also be excluded.

#### Diagnostic work-up

In addition to CT and 18F-FDG PET/CT, diffusion and dynamic contrast-enhanced MRI sequences may be useful to ensure an exhaustive assessment of the disease, particularly its cardiovascular extent. Moreover, these sequences may point out the optimal areas to biopsy (Fig. 14) [18].

Due to the histological complexity of these various entities, cytological sampling with fine-needle aspirations should be avoided and surgical biopsy sampling is often required to ensure sufficient material for detailed histologic analysis, immunohistochemical staining, molecular analyses, and culturing [11], if needed (Fig. 15) [12].

In all cases, a systematic analysis of the pulmonary veins and arteries, as well as the tracheobronchial tree and esophagus, is advised when encountering any infiltrative soft tissue density within the mediastinum. This may help to suggest the diagnosis of chronic mediastinitis and, thus, avoid delayed recognition of this condition. Such an approach has to be combined with an overall analysis of the retroperitoneum, liver, thyroid, and pancreas.

## Conclusion

In conclusion, non-malignant diffuse mediastinal disease presents with various characteristics, depending on the mechanism involved. The differential diagnosis calls for a systematic approach that includes the patient’s history and habits, excludes a tracheobronchial or esophageal injury in acute conditions, and requires a careful and precise histological examination in diffuse mediastinal tissue infiltration.

## Data Availability

All data generated or analyzed during this study are included in this published article.

## Abbreviations

18F-FDG: 2-Deoxy-2-[18F] fluorodeoxyglucose
COPD: Chronic obstructive pulmonary disease
CT: Computed tomography
FM: Fibrosing mediastinitis
PET/CT: Positron emission tomography/computed tomography
